# Prehospital guidelines on in-water traumatic spinal injuries for lifeguards and prehospital emergency medical services: an international Delphi consensus study

**DOI:** 10.1186/s13049-024-01249-3

**Published:** 2024-08-23

**Authors:** Niklas Breindahl, Joost L. M. Bierens, Sebastian Wiberg, Roberto Barcala-Furelos, Christian Maschmann

**Affiliations:** 1 Prehospital Center Region Zealand, Ringstedgade 61, 13, 4700 Næstved, Denmark; 2 Department of Neonatal and Pediatric Intensive Care, Copenhagen University Hospital, Rigshospitalet, Blegdamsvej 9, 2100 Copenhagen, Denmark; 3 Department of Clinical Medicine, University of Copenhagen, Copenhagen, Denmark; 4 International Life Saving Federation, Leuven, Belgium; 5 International Drowning Researchers’ Alliance, Kuna, ID USA; 6 Extreme Environments Laboratory, School of Sport, Health and Exercise Science, University of Portsmouth, Portsmouth, UK; 7 Department of Cardiothoracic Anaesthesiology, Copenhagen University Hospital Rigshospitalet, Copenhagen, Denmark; 8 REMOSS Research Group, Faculty of Education and Sports Sciences, Universidade de Vigo, Pontevedra, Spain; 9 Department of Emergency Medicine NFZ, Cantonal Hospital St. Gallen, Gallen, Switzerland

**Keywords:** Delphi, Guideline, Lifeguard, Emergency medical service (EMS), Drowning, Water, Spinal cord injuries, Spinal fractures, Spinal injuries, Trauma

## Abstract

**Background:**

Trauma guidelines on spinal motion restriction (SMR) have changed drastically in recent years. An international group of experts explored whether consensus could be reached and if guidelines on SMR performed by trained lifeguards and prehospital EMS following in-water traumatic spinal cord injury (TSCI) should also be changed.

**Methods:**

An international three-round Delphi process was conducted from October 2022 to November 2023. In Delphi round one, brainstorming resulted in an exhaustive list of recommendations for handling patients with suspected in-water TSCI. The list was also used to construct a preliminary flowchart for in-water SMR. In Delphi round two, three levels of agreement for each recommendation and the flowchart were established. Recommendations with strong consensus (≥ 85% agreement) underwent minor revisions and entered round three; recommendations with moderate consensus (75–85% agreement) underwent major revisions in two consecutive phases; and recommendations with weak consensus (< 75% agreement) were excluded. In Delphi round 3, the level of consensus for each of the final recommendations and each of the routes in the flowchart was tested using the same procedure as in Delphi round 2.

**Results:**

Twenty-four experts participated in Delphi round one. The response rates for Delphi rounds two and three were 92% and 88%, respectively. The study resulted in 25 recommendations and one flowchart with four flowchart paths; 24 recommendations received strong consensus (≥ 85%), and one recommendation received moderate consensus (81%). Each of the four paths in the flowchart received strong consensus (90–95%). The integral flowchart received strong consensus (93%).

**Conclusions:**

This study produced expert consensus on 25 recommendations and a flowchart on handling patients with suspected in-water TSCI by trained lifeguards and prehospital EMS. These results provide clear and simple guidelines on SMR, which can standardise training and guidelines on SMR performed by trained lifeguards or prehospital EMS.

**Supplementary Information:**

The online version contains supplementary material available at 10.1186/s13049-024-01249-3.

## Introduction

Traumatic spinal cord injury (TSCI) is defined as damage to the spinal cord following external physical impact [1]. A primary spinal cord injury happens as a result of the initial mechanical injury [2, 3]. Following the initial mechanical injury [4], a secondary spinal cord injury may be caused by vascular and biochemical effects [5, 6] such as haemorrhage [7, 8] and swelling at the site of injury into the spinal cord. Inept handling may also lead to secondary injury, and guidelines on spinal motion restrictions (SMR) are aimed at preventing this by handling patients with care. The guidelines on SMR of adult trauma patients have changed drastically in recent years [9–11]. The meaningful changes in on-land SMR fuelled the need to explore the implications for in-water SMR after in-water TCSI.

In-water TSCI most commonly occurs because of axial loading, resulting in compression of the relatively fragile cervical spine between the rapidly decelerating head and the continued momentum of the body [2, 3]. Common high-risk situations resulting in in-water TCSI are a poorly executed dive into a shallow body of water or wave-forced impacts typically occurring at moderate to severe shore breaks. Observational studies report a prevalence of spinal fractures from diving accidents of approximately 10% of the total population admitted with TSCI [12–18]. In-water TSCI typically occurs in young, healthy males under 30 who sustain no other associated intracranial or systemic injuries. Most spinal cord injuries in swimming pools result from reckless behaviour [19], involvement of alcohol [20], a lack of warning signs or depth indicators [20], and no lifeguard on duty. [20]

The most common levels of injury are C-5 and C-6 [13, 21], The rate of neurological injuries such as paralysis and sensory deficits following in-water TSCI is high and varies between 22 and 90%. [2, 12, 13, 17, 18].

In-water TSCI is a rare and complicated situation for trained lifeguards and prehospital EMS [22]. No standard exists, and various procedures are used worldwide [19].

This study aimed to establish international expert consensus on handling patients with suspected in-water TSCI to standardise guidelines on SMR performed by trained lifeguards and prehospital EMS.

## Materials and methods

### Study design

A Delphi process is a well‐established, systematic, consensus‐building method for collecting expert opinions and achieving agreement when objective information is unavailable [23]. We conducted a modified Delphi process with international participation. The study used three iterative rounds of online survey questionnaires, including structured and semi-structured questions: Delphi round 1 (brainstorm), Delphi round 2 (consensus), and Delphi round 3 (approval). The study was conducted in adherence with a detailed prespecified protocol available from the corresponding author upon request and is reported in compliance with the ACcurate COnsensus Reporting Document (ACCORD) [24] (Online Supplement, Appendix A).

### Steering committee

Before the study started, a steering committee was installed to manage all the steps in the modified Delphi process, including drafting the invitations to participate and the first version of the recommendations, developing and pretesting survey questionnaires, and adapting the recommendations and the flowchart based on experts’ comments. The steering committee included a multi-national and multi-professional team experienced in medical research, prehospital and emergency medicine, spinal trauma management, and lifeguarding. The steering committee members were not allowed to participate as experts in the consensus process during the Delphi rounds.

### Sample characteristics

Strict criteria were defined to select experts competent to establish consensus on recommendations for handling patients with suspected in-water TSCI. Members of the International Drowning Researchers’ Alliance (IDRA), the International Life Saving Federation (ILS) Medical Committee (ILS-MC), and the ILS Rescue Commission (ILS-RC) were regarded as eligible for inclusion as potential experts.

The inclusion criteria for these members included a background in clinical health care as a medical doctor, nurse, paramedic/EMT or similar and at least one of the following three criteria: (1) Having clinical expertise in handling patients with suspected in-water TSCI, (2) Having teaching expertise in handling patients with suspected in-water TSCI, (3) Having research expertise on in-water TSCI. We aimed for approximately 23 participants, as other research findings suggest that that number of participants led to response stability during multiple Delphi rounds [25].

### Survey administration

The secretaries from ILS-MC, ILS-RC, and IDRA emailed the invitations to their members. The invitation included background information outlining the purpose of the study, the importance of participation, and a link to the survey questionnaire for Delphi round 1. All responses in Delphi Round 1 generated a unique participant identification number in REDCap, which was used to send personal links during rounds 2 and 3, securing anonymity and preventing multiple participation. All communication between the experts and the primary investigator (NB) was conducted through email.

The answers to the surveys could be saved at any time by the experts, allowing them to access and edit their answers later until reaching the deadline. Non-respondents received deadline reminders every week until the deadline. After the deadline, access to the survey was closed to ensure the progression and termination of the study*.*

### Data collection methods

Data were systematically collected through all Delphi rounds using the Research Electronic Data Capture (REDCap) system [26]. The predefined minimum number of experts to start the study was 20 participants. The risk of non-response error was minimised through weekly deadline reminders highlighting the importance of participation and providing a deadline extension. Experts who failed to answer before the extended deadline were excluded from the following rounds.

### Delphi round 1 (brainstorm)

In Delphi round 1, the experts were asked to provide information about their sex, age, country, affiliations, local practices, and contact information. After completing these data, they were guided to a summary of the existing literature on in-water TSCI, including three questions to check their understanding of the current knowledge produced by the primary author (Online Supplement, Appendix B). Once the experts had answered the three questions correctly, they were asked to comment with free text on the 30 recommendations suggested by the steering committee. All recommendations were clarified by a rationale, including references to publications providing supporting arguments for some of the recommendations.

Following Delphi round 1, the steering committee adapted the recommendations based on the experts' comments, removed duplicates, and identified various textual expressions for each unique recommendation to consolidate the list of recommendations. The steering committee could change the wording if the meaning was preserved. Decisions were based on unanimous agreement among the steering committee members. The steering committee also constructed a preliminary flowchart for managing in-water TSCI based on the preliminary recommendations following Delphi round 1.

### Delphi round 2 (consensus)

In Delphi round 2, the consensus level for the adapted recommendations and the flowchart were tested. The experts replied to the following question: “*How much do you agree with the following recommendation/flowchart?*” The experts could indicate their agreement with each recommendation on a 4-point Likert Scale: (1) Strongly disagree, (2) Disagree, (3) Agree, and (4) Strongly agree. The experts were urged to explain their ratings. The consensus levels were calculated as the combined frequencies of “agree” and “strongly agree". Three categories were defined: (1) Strong consensus with unanimous or almost unanimous agreement (≥ 85%), (2) Moderate consensus with a substantial agreement (75–85%), and (3) Weak consensus with a low agreement (< 75%). Items with weak consensus were excluded in Delphi round 2.

For the recommendations with moderate consensus, adaptations were made by the steering committee based on the comments made by the experts and Delphi round 2 was repeated. Recommendations with strong consensus were marginally adapted by the steering committee, based on the comments made by the experts, and then directly passed to Delphi round 3.

The steering committee also produced a preamble based on the experts’ comments explaining some core concepts as prerequisites for the recommendations to improve the readability. According to the preamble, all recommendations presented in this study can only be performed if the scene is safe. All recommendations are contraindicated in any circumstance with imminent danger of drowning or injury (e.g., high surf, fast-moving water, or rocky areas). A "lifeguard" was defined as a person who has completed professional training, including training in handling TSCI and performing SMR and extrication, and is competent to prevent injury, perform rescues, and provide first aid to those in and around aquatic environments [27]. “Spinal motion restriction” (SMR) was defined as the procedure used on a patient with suspected TSCI to reduce spinal movement, irrespective of adjuncts or devices [27]. “Extrication” was defined as transporting the patient with suspected in-water TSCI from the water to the land using the appropriate SMR measures.

### Delphi round 3 (approval)

In Delphi round 3, the level of consensus for the final set of recommendations and the flowchart was tested. The consensus levels derived from Delphi Round 2 were unmasked, and the experts replied again to the question: “*How much do you agree with the following recommendations/flowchart?*” The identical 4-point Likert Scale was used as in Delphi round 2.

If the experts chose “disagree” or “strongly disagree” for a recommendation, they were asked to explain their ratings. The consensus levels were calculated as the combined frequencies of “agree” and “strongly agree”.

If the experts disagreed with any specific routes in the flowchart, they were asked to explain their ratings. The consensus levels were calculated as the combined frequencies of “agree” and “strongly agree” for the specific routes, and the average was used as the final consensus level. The threshold level of consensus for each route was ≥ 75% of the experts [28]. Items achieving the threshold were accepted without further adaptations.

### Statistical analysis

Categorical data from Delphi round 1 were presented as counts and percentages and numerical variables as medians with interquartile ranges [IQR] and range as appropriate. All analyses were performed using R Statistical software (R version 4.3.1 [2023-06-16 ucrt]) [29]. There was no imputation of missing data. This study did not adjust for the non-representativeness of the sample or use sensitivity analysis.

## Results

Expert demographics are summarised in Table 1. A total of 18 (75%) experts did not have a local or national guideline on handling in-water TSCI before initiating this study.

**Table 1:** Expert demographic

Variable	Experts (n = 24)
Sex, male, n (%)	21 (88)
Age, median years [IQR]	48 [42–51]
Organisation, n (%)*	
IDRA	17 (71)
ILS Medical Committee	10 (42)
ILS Rescue Commission	5 (21)
Background in clinical health care, n (%)*	
Medical Doctor	9 (38)
Registered Nurse	3 (13)
Paramedic/Emergency Medical Technician	10 (42)
Other healthcare background	6 (25)
Field of expertise with in-water TSCI, n (%)*	
Clinical expertise	19 (79)
Teaching expertise	22 (92)
Research expertise	2 (8)
No existing local/national guideline on handling in-water TSCI, n (%)	18 (75)
Country, n (%)	
3 participants: New Zealand, Spain, USA	9
2 participants: Canada, Portugal, UK	6
1 participant: Argentina, Australia, Egypt, France, Guatemala, Hong Kong, The Netherlands, Sweden, Uganda	9

A total of 24 experts participated in Delphi round 1 and answered demographic information

*Multiple choice fields

ILS, International Life Saving Federation; IDRA, International Drowning Researchers’ Alliance; IQR, Interquartile ranges; TSCI, Traumatic Spinal Cord Injury

**Fig. 1:**
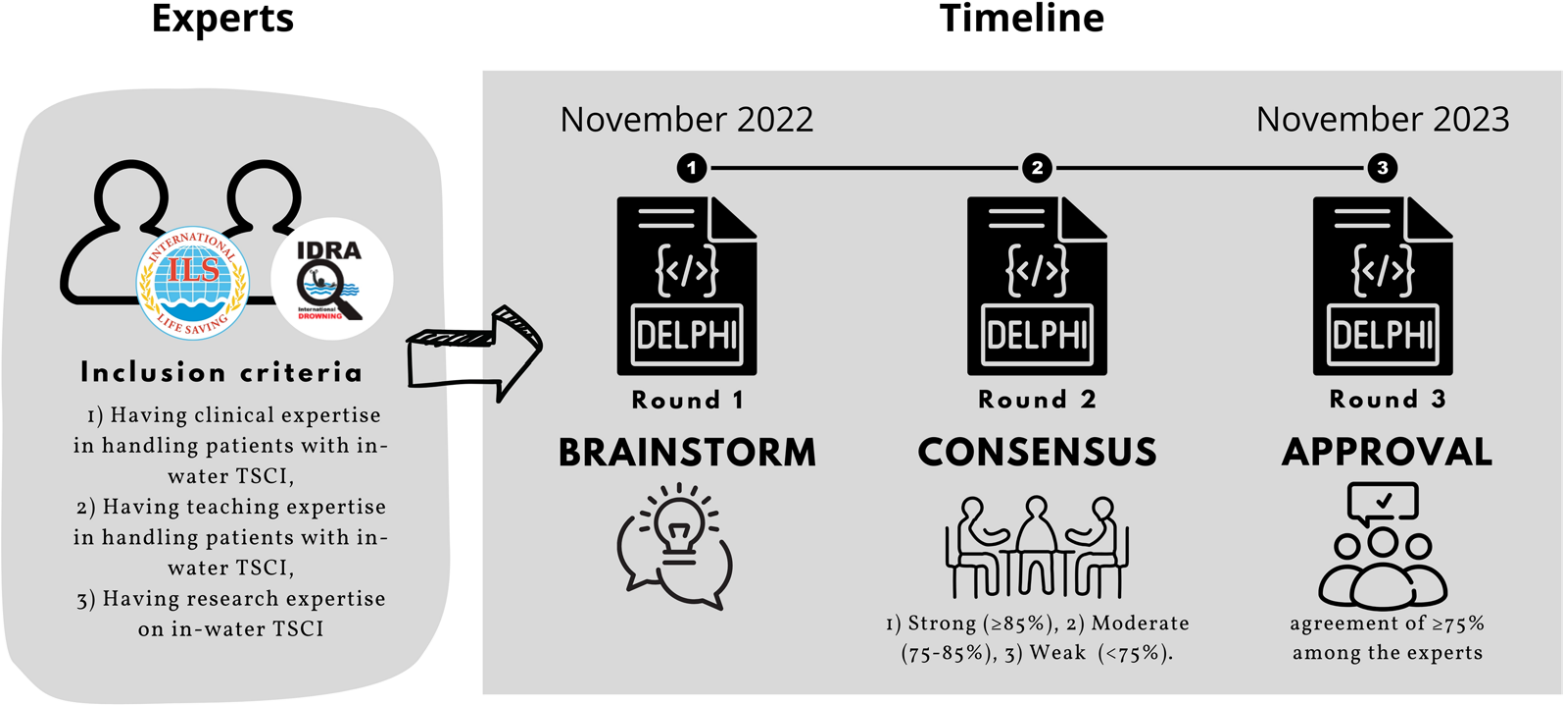
Study flow. Members of the International Life Saving Federation (ILS) Medical Committee (ILS-MC), the ILS Rescue Commission (ILS-RC), and the International Drowning Researchers’ Alliance (IDRA) with a background in clinical health care as a medical doctor, nurse, paramedic/EMT or similar were eligible for inclusion as potential experts. The inclusion criteria included at least one of the following three criteria: (1) Having clinical expertise in handling patients with suspected in-water TSCI, (2) Having teaching expertise in handling patients with suspected in-water TSCI, and (3) Having research expertise on in-water TSCI. The study was initiated in October 2022, concluded in November 2023, and consisted of three Delphi rounds. In Delphi round two, the level of agreement for each recommendation and the flowchart was calculated as the frequency of “agree” (3) and “strongly agree” (4) on a 4-point Likert-like scale and divided into three levels: (1) recommendations with strong consensus (≥ 85% agreement) underwent minor revisions and entered round three, (2) recommendations with moderate consensus (75–85% agreement) underwent major revisions and repeated round two, (3) recommendations with weak consensus (< 75% agreement) were excluded. In Delphi round 3, the level of consensus for each of the final recommendations and each of the routes in the flowchart was tested using the same procedure as in Delphi round 2. The consensus threshold was an agreement of ≥ 75% among the experts

**Fig. 2:**
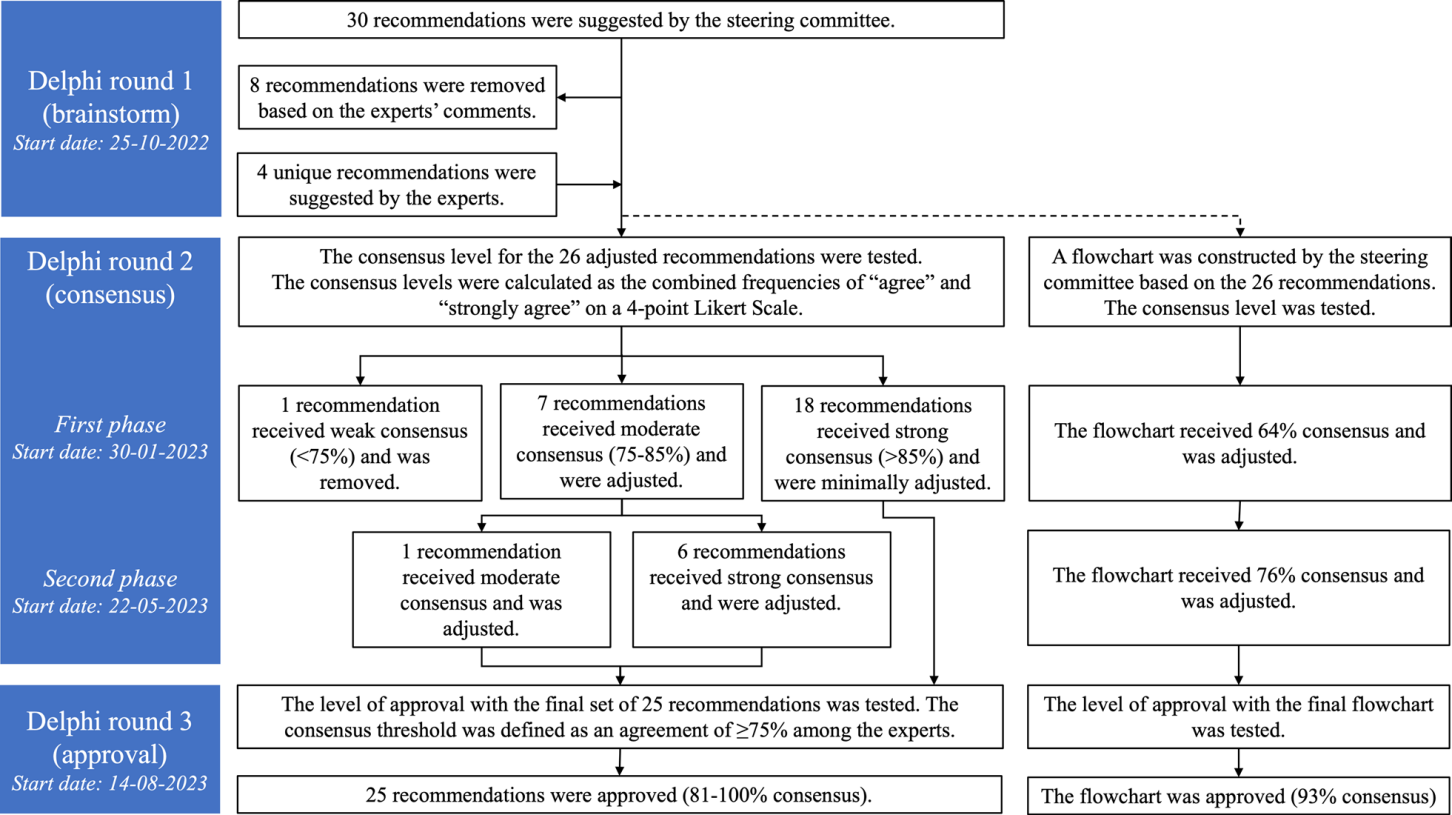
Workflow diagram of the modified Delphi study. The study was initiated in October 2022, concluded in November 2023, and consisted of three Delphi rounds. In Delphi round two, the level of agreement for each recommendation and the flowchart was calculated as the frequency of “agree” (3) and “strongly agree” (4) on a 4-point Likert-like scale and divided into three levels: (1) recommendations with strong consensus (≥ 85% agreement) underwent minor revisions and entered round three, (2) recommendations with moderate consensus (75–85% agreement) underwent major revisions and repeated round two, (3) recommendations with weak consensus (< 75% agreement) were excluded. In Delphi round 3, the level of consensus for each of the final recommendations and each of the routes in the flowchart was tested using the same procedure as in Delphi round 2. The consensus threshold was an agreement of ≥ 75% among the experts

The data were collected during the three Delphi rounds from October 2022 to November 2023 (Fig. 1). A detailed workflow diagram is available in Fig. 2.

In Delphi round 1, 30 recommendations were suggested by the steering committee. Based on the experts’ comments, 8 recommendations were removed, and 4 recommendations were added. Based on the 26 recommendations, the steering committee constructed a flowchart.

In the first phase of Delphi round 2, 22/24 experts responded (92%). One recommendation received weak consensus and was removed. Seven recommendations received moderate consensus, and 18 recommendations received strong consensus. The recommendations with moderate consensus were adapted and passed to the second phase of Delphi round 2.

In the second phase of Delphi round 2, 21/24 experts responded (88%). One recommendation received moderate consensus, and six recommendations received strong consensus. A total of 25 recommendations were passed to Delphi round 3: 24/25 recommendations with strong consensus and 1/25 recommendations with moderate consensus. A complete list of recommendations and consensus levels from Delphi rounds 2 and 3 are available (Online Supplement, Appendix C).

The flowchart received weak consensus (64%) in the first phase of Delphi round 2 and was adapted. In the second phase of Delphi round 2, the flowchart received moderate consensus (76%) and was adapted bases on the comments received before being passed to Delphi round 3.

In Delphi round 3, 21/24 experts responded (88%). All 25 recommendations were individually approved, with a consensus of 81–100% (Table 2). The only recommendation with a moderate level of consensus was: “*It is recommended to use at least three persons to perform spinal motion restriction to extricate a patient suspected of in-water traumatic spinal cord injury. At least one person should be specifically trained. If the necessary number of persons is not available, do not further delay extrication.*” All other recommendations received a strong consensus level. The final set of 25 recommendations was divided into four sections: (1) a pre-rescue section consisting of five recommendations, (2) a rescue section consisting of 14 recommendations, (3) a post-rescue section consisting of two recommendations, and (4) a patient selection section consisting of four recommendations.

**Table 2:** The final set of recommendations clarified by a rationale with levels of agreement

Recommendation	Rationale	Final agreement
*Pre-rescue section*		
**R1:** It is recommended against using spinal motion restriction when the trauma is due to a mechanism unlikely to cause spinal cord injury	Patients are at risk for in-water traumatic spinal cord injury only if they have also sustained a relevant trauma. Spinal motion restriction should not be used solely based on a history of drowning because it is time-demanding and may delay the rescue [30]	95%
**R2:** It is recommended to always alert the available EMS, but this should not delay the rescue	The mechanism of injury related to in-water traumatic spinal cord injury, such as diving into a shallow body of water or wave-forced impacts, may cause severe intracranial haemorrhage, spinal cord injury, fractures, or bleedings. Early transportation to the hospital and definitive treatment is imperative. Current guidelines on spinal motion restriction of suspected traumatic spinal cord injury recommend calling for an ambulance as one of the top priorities [31]	100%
**R3:** It is recommended to assess scene safety before attempting a water rescue. This assessment should include the aquatic conditions (such as high surf, fast-moving water, or rocky areas), the level of training and the experience of the lifeguards, the number of lifeguards available and needed, the size of the patient, and the equipment available	The safety of the lifeguard(s) and the patient(s) must always be the top priority	100%
**R4:** It is recommended against using spinal motion restriction in patients suspected of in-water traumatic spinal cord injury in any circumstance with imminent danger of drowning or injury to the lifeguard	Spinal motion restriction should only apply to situations where the scene is safe. The lifeguard should prioritise a fast rescue of patients suspected of in-water traumatic spinal cord injury who are in imminent danger of drowning or injury (e.g., high surf, fast-moving water, or rocky areas). Spinal motion restriction in these locations will increase the risk to the lifeguard(s) and patient(s)	100%
**R5:** It is recommended against using spinal motion restriction in patients suspected of in-water traumatic spinal cord injury who are unconscious and not breathing normally (suspected cardiac arrest)	Assessment of consciousness and breathing in the water may be difficult. If the lifeguard is unsure, the patient should be treated as if the patient was in cardiac arrest, requiring resuscitation as soon as possible and urgent transfer to the hospital	95%
*Rescue section*		
**R6:** It is recommended to turn a face-down patient suspected of in-water traumatic spinal cord injury immediately and carefully into a face-up position	Any patient face-down in the water is in imminent danger of hypoxia or drowning and must be turned face-up immediately to assess the level of consciousness and breathing. Various techniques are currently instructed to turn a face-down patient. Importantly, lifeguards must secure a stable position of the head in relation to the thorax during the turn	90%
**R7:** It is recommended to use the AVPU scale to identify an altered level of consciousness in patients suspected of in-water traumatic spinal cord injury	The AVPU (Alert, Verbally responsive, Painfully responsive, Unresponsive) scale is a fast and simple way of detecting an altered level of consciousness in patients, even during a rescue. It is feasible in the prehospital setting as a score lower than “A and oriented” should be considered abnormal until proven otherwise [32, 33]	95%
**R8:** It is recommended to use the symptom of spinal pain to assess the need for spinal motion restriction in alert patients without a critical ABC problem suspected of in-water traumatic spinal cord injury by asking: “Do you feel pain in your neck or back?”	Lifeguards are not trained healthcare professionals, and clinical assessments in the aquatic environment are challenging. Simple and sensitive diagnostic tools should guide clinical decision-making. Current guidelines on spinal motion restriction of trauma patients recommend using spinal pain to assess the need for spinal motion restriction in alert patients suspected of in-water traumatic spinal cord injury [9]	90%
**R9:** It is recommended to use obvious signs of any neurological deficit to assess the need for spinal motion restriction in alert patients without a critical ABC problem suspected of in-water traumatic spinal cord injury by asking: “Can you move your arms and legs?”	Lifeguards are not trained healthcare professionals, and clinical assessments in the aquatic environment are challenging. Hence, simple and sensitive diagnostic tools should guide clinical decision-making. Current guidelines on spinal motion restriction of trauma patients recommend using neurological deficits to assess the need for spinal motion restriction in alert patients suspected of in-water traumatic spinal cord injury [9]	90%
**R10:** It is recommended to use spinal motion restriction for extrication in alert and oriented patients without a critical ABC problem suspected of in-water traumatic spinal cord injury where self-extrication is impossible	If the patient is alert, oriented, and suspected of in-water traumatic spinal cord injury, the lifeguard should ask, “Can you stand up?". Current guidelines on spinal motion restriction of trauma patients recommend spinal motion restriction for extrication of alert and oriented trauma patients where self-extrication is impossible [9]	90%
**R11:** It is recommended against using a rigid cervical collar in all patients suspected of in-water traumatic spinal cord injury	Based on recent research, current guidelines on spinal motion restriction of trauma patients recommend against using a rigid cervical collar as there are no proven benefits on neurological outcomes or mortality [10, 34–40], and the effect on the range of motion in the cervical spine is very limited [37, 41–44]. Furthermore, using a rigid cervical collar is correlated to a series of harmful effects such as impeded airway management [10], worsening of existing cervical injury [10], increased spinal movement due to pain or discomfort [10], elevation of intracranial pressure due to impeded venous drainage through the neck [45, 46], and prolonged stay in the emergency room [47]	95%
**R12:** It is recommended to in-line stabilise the head in relation to the thorax with two hands during the extrication if in-water traumatic spinal cord injury is suspected	If the patient cannot perform self-extrication, lifeguards must perform spinal motion restriction and extrication from the water. Various techniques are currently instructed to manually stabilise the patient’s head during the extrication. Importantly, lifeguards must be trained in a technique that secures a stable position of the head in relation to the thorax during the extrication and is suitable for the specific circumstance	86%
**R13:** It is recommended to use a floatable, lightweight device that drains water and is appropriate to water conditions to perform spinal motion restriction for extrication of alert patients suspected of in-water traumatic spinal cord injury who cannot perform self-extrication	Various boards and stretchers have been approved for handling a patient suspected of in-water traumatic spinal cord injury, and these may be an appropriate solution for the given situation	95%
**R14:** It is recommended against using straps in water unless required for safe extrication	Using straps in the water can be dangerous as it may cause situations in which water aspiration, submersion, or permanent loss can occur. The in-water use of straps is often time-consuming and inefficient. However, in some circumstances (e.g., related to pool designs), extrication requires straps to prevent the patient from being dropped or sliding off the board	90%
**R15:** It is recommended that one lifeguard trained in spinal motion restriction acts as the team leader and is responsible for the stabilisation of the patient's head, the team's safety, supervision, instructions, and coordination	Lifeguards performing spinal motion restriction need to be sure that they maintain spinal alignment during extrication and transportation without risks to the rescuers or the patient. Therefore, a team leader responsible for the team’s safety, supervision, instructions, and coordination should be appointed. The team leader is responsible for the stabilisation of the patient's head	100%
**R16:** It is recommended to use at least three persons to perform spinal motion restriction to extricate a patient suspected of in-water traumatic spinal cord injury. At least one person should be specifically trained. If the necessary number of persons is not available, do not further delay extrication	Various extrication techniques are currently instructed. All these techniques require a minimum of three persons to be performed successfully with minimal risk to the patient. At least one of the lifeguards should be trained in spinal motion restriction	81%
**R17:** It is recommended to integrate untrained bystanders under the leadership of the lifeguard(s) if there are not enough trained lifeguards available for spinal motion restriction and extrication	Untrained bystanders can be asked to support the lifeguard if the required number of lifeguards are not available to perform spinal motion restriction. This may improve the quality of spinal motion restriction and lower the risks to the lifeguard(s) and the patient(s)	90%
**R18:** It is recommended against using spinal motion restriction in patients suspected of in-water traumatic spinal cord injury who have NO relevant symptoms	Traumatic spinal cord injury will cause symptoms. These symptoms must guide the spinal motion restriction. If the patient has NO relevant symptoms (no spinal pain and normal movement in arms and legs), in-water traumatic spinal cord injury should not be suspected, and spinal motion restriction should not be performed. Current guidelines on spinal motion restriction of trauma patients recommend no spinal motion restriction in asymptomatic trauma patients because (1) Significant spinal injury is unlikely to occur without causing any symptoms [9], and (2) Numerous studies have demonstrated possible hazardous effects of spinal stabilisation including pain [36, 40, 48, 49], development of pressure ulcers [48–50], difficult clinical examination [36], and prolonged prehospital on-scene time [48]	95%
**R19:** It is recommended to use self-extrication and self-stabilisation in alert patients suspected of in-water traumatic spinal cord injury who can perform self-extrication and self-stabilisation	The risk of an unstable spinal injury in alert patients is rare [34]. Additionally, alert patients will automatically stabilise their spine in the most comfortable position [9, 34, 51]. Current guidelines on spinal motion restriction of trauma patients recommend encouraging an alert trauma patient to self-stabilise the spine and perform self-extrication [32]. Lifeguards should always help the patient perform self-stabilisation (e.g., support the patient so that the risk of falling or stumbling is reduced as much as possible, protect from waves, remove stones and other submerged objects)	95%
*Post-rescue section*		
**R20:** It is recommended to allow an alert patient to place him/herself in the most comfortable position during spinal motion restriction on land	Alert patients will automatically stabilise their spine in the most comfortable position [9, 34, 51]. Current guidelines on spinal motion restriction of trauma patients recommend encouraging an alert trauma patient to self-stabilise the spine and focusing on optimising patient comfort [32]. Alert patients should be allowed to sit or lay down comfortably until EMS professionals arrive	95%
**R21:** It is recommended to use the jaw thrust manoeuvre with the head in a neutral position to open the airway in patients suspected of in-water traumatic spinal cord injury who cannot maintain an open airway	Airway management should always be a top priority for all patients with suspected spinal injuries. Various techniques for airway management exist. If a neutral head position does not open the airway, use the jaw thrust manoeuvre before the head tilt [52]	95%
*Patient selection section*		
**R22:** It is recommended to treat potentially intoxicated patients in the same way as non-intoxicated patients suspected of in-water traumatic spinal cord injury	It is often not possible to rule out or diagnose intoxication clinically since it is difficult to differentiate, for example, between intoxication symptoms, concussions, or critical neurological injuries (e.g., intracranial haemorrhage [53]). Current guidelines on spinal motion restriction of trauma patients recommend that trauma patients being affected by alcohol or drugs be treated in the same way as all other non-intoxicated trauma patients [9]	86%
**R23:** It is recommended to treat patients with distracting injuries in the same way as patients without distracting injuries suspected of in-water traumatic spinal cord injury	Distracting injuries do not disturb the sensitivity of a spine examination [54–56] Current guidelines on spinal motion restriction of trauma patients recommend treating trauma patients with distracting injuries in the same way as all other trauma patients [9]	86%
**R24:** It is recommended to treat patients with language barriers in the same way as patients without language barriers suspected of in-water traumatic spinal cord injury	Language barriers will challenge history taking, but current guidelines on spinal motion restriction of trauma patients recommend treating trauma patients with language barriers in the same way as all other alert trauma patients [57]. The lifeguard may assess spinal pain by interpreting the patient's facial expressions and neurological deficits by observing the patient's spontaneous movements in arms and legs	90%
**R25:** It is recommended to treat children in the same way as adults suspected of in-water traumatic spinal cord injury	Children with suspected in-water traumatic spinal cord injury should be treated in the same way as adults, including stabilisation with two hands (bimanual stabilisation) [52]. Children under eight may require an additional 2.5 cm back elevation under their shoulders to achieve a better neutral head position in the supine position [58]	90%

The final set of 25 recommendations was divided into four sections: (1) the pre-rescue section consisting of five recommendations, (2) the rescue section consisting of 14 recommendations, (3) the post-rescue section consisting of two recommendations, and (4) the patient selection section consisting of four recommendations. The experts indicated their agreement with each of the recommendations on a 4-point Likert Scale: (1) Strongly disagree, (2) Disagree, (3) Agree, and (4) Strongly agree. The levels shown were calculated as the combined frequencies of “agree” and “strongly agree”. A total of 24 recommendations received a strong consensus level (≥ 85%), and only one recommendation (R16) received a moderate consensus level (81%)

**Fig. 3:**
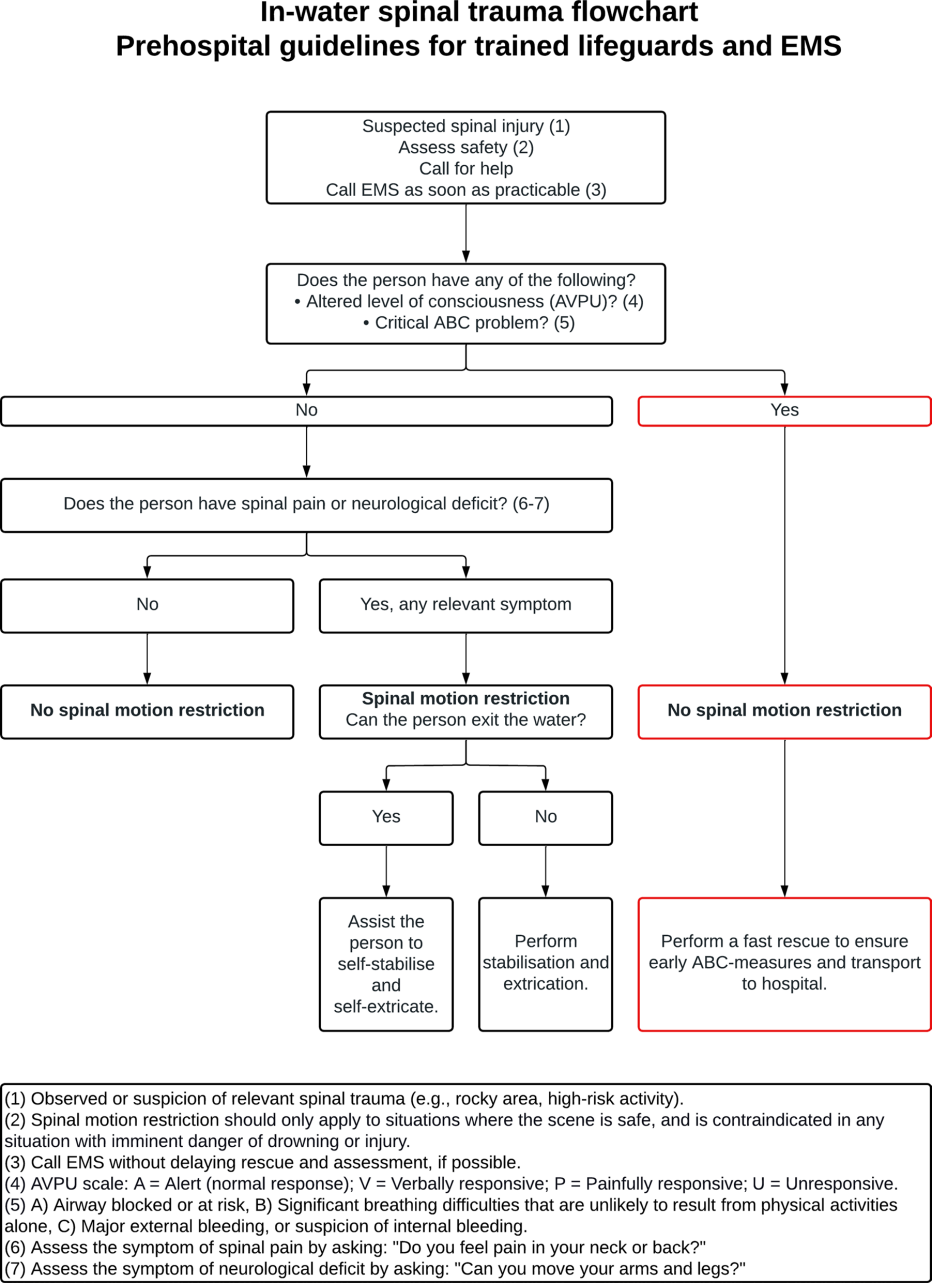
In-water spinal trauma flowchart—prehospital guidelines for trained lifeguards and prehospital EMS. The flowchart was constructed and adjusted according to the recommendations. The flowchart received strong consensus (93%) in Delphi round 3. Each of the four routes in the flowchart received strong consensus: Route 1 to the left (90%), route 2 in the middle left (95%), route 3 in the middle right (95%), and route 4 to the right (90%). Footnotes are provided and should be used together with the flowchart

The final flowchart (Fig. 3) received a strong level of consensus with an overall agreement of 93%. Each of the four routes in the flowchart received a strong level of consensus (90–95%) (Table 3).

**Table 3:** Flowchart with levels of agreement

**Delphi round 2, phase 1**				
Weak consensus (64%)				
**Delphi round 2, phase 2**				
Moderate consensus (76%)				
**Delphi round 3**				
**Route 1**	**Route 2**	**Route 3**	**Route 4**	**Average**
Strong consensus, 19/21 (90%)	Strong consensus, 20/21 (95%)	Strong consensus, 20/21 (95%)	Strong consensus, 19/21 (90%)	Strong consensus, 93%

The experts indicated their agreement with the flowchart on a 4-point Likert Scale: (1) Strongly disagree, (2) Disagree, (3) Agree, and (4) Strongly agree. The levels shown were calculated as the combined frequencies of "agree" and "strongly agree". Three categories were defined: (1) Strong consensus (≥ 85%), (2) Moderate consensus (75–85%), and (3) Weak consensus (< 75%). In Delphi round 3, each of the four routes in the flowchart received a strong consensus (90–95%), resulting in an overall strong consensus of 93%

## Discussion

A total of 24 experts from 15 countries participated in this study to produce an international expert consensus on handling patients with suspected in-water TSCI. Eighteen (75% of the experts) did not have a local or national guideline on handling in-water TSCI before the initiation of this study emphasizing the need for development of guidelines. This study produced a list of 25 recommendations and a flowchart to standardise guidelines on SMR performed by trained lifeguards or prehospital EMS of patients with suspected in-water TSCI. All 25 recommendations were individually approved, with a consensus of 81–100%. The final flowchart received a strong level of consensus with an overall agreement of 93%. Each of the four routes in the flowchart received a strong level of consensus (90–95%).

### Consistency with the existing literature

EMS systems worldwide use different triaging tools to decide whether to perform SMR [10, 59–61]. The recommendations and flowchart developed in this study have many similarities with recent Scandinavian guidelines, adding to the external validity of our findings [9–11]. One major exception is for patients with an altered level of consciousness or a critical ABC problem, where “Time-critical spinal motion restriction” has been replaced with “No spinal motion restriction”, as attempting to perform SMR on these groups of patients in the water may significantly increase the risk of drowning. The same applies to circumstances with imminent danger of drowning or injury, as performing SMR in locations with high surf, fast-moving water, or rocky areas will increase the risk to the lifeguard(s) and patient(s). Other triaging tools are based on decision aids like the Canadian C-Spine Rule (CCSR) [62], or the National Emergency X-radiography Utilisation Study (NEXUS) [63]. These decision aids were initially intended to decide whether the patient needed radiography and were later extrapolated as a decision aid on SMR [64]. The NEXUS rule addresses intoxication and distracting injuries specifically. The focus on intoxication and distracting injuries were removed in the Scandinavian guidelines [9]. It is impossible to rule out intoxication clinically and difficult to differentiate between intoxication symptoms, concussions, or critical neurological injuries [9, 53], and studies indicate that distracting injuries do not disturb the sensitivity of a spine examination [54–56].

During the Delphi process, some issues enriched the understanding of what makes in-water TSCI special.

Any patient face-down in the water is in imminent danger of hypoxia or drowning and must be turned face-up immediately and carefully. The word “carefully” highlights the need for securing a stable position of the head in relation to the thorax during the turn, as inept handling of these patients may also lead to secondary injury. However, this must not cause delay.

Clinical assessments in the aquatic environment are challenging, and lifeguards are not trained as healthcare professionals to perform clinical examinations. Simple and sensitive diagnostic tools should guide clinical decision-making. We recommend using the symptoms of spinal pain and neurological deficits to assess the need for spinal motion restriction in alert patients without a critical ABC problem suspected of in-water TSCI by asking the patient: “Do you feel pain in your neck or back?” and “Can you move your arms and legs?”. Once on land, EMS personnel should perform additional assessments of the patient as part of advanced patient care.

Alert patients suspected of in-water TSCI who can perform self-stabilisation and self-extrication should be guided to do so [9, 32], as the risk of an unstable spinal injury in alert patients is extremely low [34], and alert patients will automatically stabilise their spine in the most comfortable position [9, 34, 51]. This should also be the case for children [52], allowing them to sit with their parents when possible. Alert patients, including children without a critical ABC problem who cannot perform self-stabilisation and self-extrication, should be extricated using SMR. Children under eight may require an additional 2.5 cm back elevation under their shoulders to achieve a better neutral head position in the supine position [58]. Guiding self-extrication, performing SMR, and placing back elevation in young children require specific training. Therefore, we defined a “lifeguard” in the preamble as someone who has completed professional training in SMR and extrication.

Using at least three persons to perform SMR achieved consensus among the experts. However, depending on the local circumstances and availability of lifeguards, SMR can be practised with fewer lifeguards. We also recommended integrating untrained bystanders under the leadership of the lifeguard if there are not enough trained lifeguards available. This could delay extrication yet improve the quality of spinal motion restriction and lower the risks to the lifeguard and the patient and may be used in specific situations.

### Strengths

This study has several strengths. We used purposive sampling to select a suitable group of experts with the necessary expertise. We used clear expert inclusion criteria to avoid introducing bias and sent invitations to the ILS-MC, ILS-RC, and IDRA. The experts represented high-, low-, and middle-income countries from Europe, North America, South America, Asia, Africa, and Oceania, adding to the generalizability of our findings. The Delphi rounds 2 and 3 achieved high response rates, limiting the risk of non-response bias. The risk of group pressure, frequently associated with expert panels, was minimised by providing a unique link for each expert per round and anonymising all responses before analyses [23, 65].

Finally, lifeguards worldwide spend considerable time practising complicated SMR techniques and extrication from challenging aquatic environments, believing that these techniques may prevent secondary injury [19]. This study provides international prehospital standards on handling in-water TSCI, which can be used to uniformise lifeguard training.

### Limitations

This study has several limitations. The broad inclusion criteria might have diluted the qualification of being an “expert”. However, data showed that 79% of the experts had clinical expertise, and 92% had teaching expertise with in-water TSCI. Conversely, trained lifeguards, EMS personnel, patients, and the public were underrepresented or absent from the study. Future research should obtain the views of more diverse stakeholder groups. The steering committee may have gained influence and introduced confirmation and acquiescence bias by summarising the existing body of evidence and suggesting a preliminary set of recommendations in Delphi round 1, which was based on the recent Scandinavian guidelines [9]. This seems unlikely as the decision to remove or rephrase the recommendations was based exclusively on the experts’ opinions and was not in any way influenced by the steering committee. Also, the high levels of agreement in the subsequent Delphi rounds make this influence unlikely.

The scarcity of high-quality evidence regarding in-water TSCI is a significant limitation to developing clinical guidelines, including recommendations for or against certain types of equipment (e.g., backboard). For now, it remains unlikely that well-designed, prospective studies, including randomised clinical trials focusing on the aquatic environment, are possible. Despite the low-quality evidence supporting these guidelines, the recommendations and the flowchart can serve as the best standard for a useful decision aid for trained lifeguards and prehospital EMS. These guidelines provide a simple and realistic method for SMR which can be implemented in lifeguard training programs to reduce unnecessary time expenditure while maximising the lifeguards’ level of competency.

However, caution is needed in implementing some of the recommendations, as there may be legal issues regarding equipment use.

## Conclusion

This study produced international expert consensus on 25 recommendations and a flowchart on handling patients with suspected in-water TSCI. These simple guidelines provide a feasible and structured approach to perform SMR of patients with suspected in-water TSCI and can serve to standardise lifeguard training, patient care, and cooperation with prehospital EMS.

### Supplementary Information

Additional file 1.

Additional file 2.

Additional file 3.

Additional file 4.

## Data Availability

The survey questionnaires and datasets used and analysed during the current study are available from the corresponding author upon reasonable request.

## Abbreviations

ACCORD: ACcurate COnsensus Reporting Document
EMS: Emergency medical service
IDRA: International Drowning Researchers’ Alliance
ILS: International Life Saving Federation
ILS-MC: International Life Saving Federation Medical Committee
ILS-RC: International Life Saving Federation Rescue Commission
IQR: Interquartile ranges
REDCap: Research Electronic Data Capture
SMR: Spinal motion restriction
TSCI: Traumatic spinal cord injury
