# Supplementary material for: Prehospital guidelines on in-water traumatic spinal injuries for lifeguards and prehospital emergency medical services: an international Delphi consensus study
Source: Scand J Trauma Resusc Emerg Med. 2024 Aug 23;32:76. doi: 10.1186/s13049-024-01249-3 (PMC11344453; doi:10.1186/s13049-024-01249-3)
Supplement: Supplementary file 2 — Additional file 2. [file 13049_2024_1249_MOESM2_ESM.docx]

**Online Supplement, Appendix B.** Summary of the existing literature on in-water TSCI before initiation of the study provided to the experts in Delphi round 1

**Summary of the existing literature:**

*Traumatic spinal cord injury (TSCI) is defined as damage to the spinal cord following an external physical impact. Submersion patients are at risk for cervical spine injury only if they have also sustained a traumatic injury, and routine spinal motion restriction (SMR) should not be used solely based on a history of submersion. In-water TSCI most commonly occur because of axial loading resulting in compression of the relatively fragile cervical spine between the rapidly decelerating head and the continued momentum of the body. Common high-risk situations are a poorly executed dive into a shallow body of water where the patient strikes his head on the bottom or hidden submerged objects, or wave-forced impacts of the head with the ocean bottom typically occurring at moderate to severe shore breaks. A primary spinal cord injury happens with the initial mechanical injury while secondary spinal cord injury following the mechanical injury may be caused by vascular and/or biochemical effects such as haemorrhage and swelling at the site of injury into the spinal cord.*

*Observational studies report a prevalence of spinal fractures from diving accidents of approximately 10% of the total population admitted with TSCI. Despite the rarity of TSCI the consequences can be devastating. The most common levels of injury are C-5 and C-6, which may cause disabling neurological injury including paralysis and sensory deficits. The rate of neurological injuries following in-water TSCI is high and varies between 22-90%. Subsequent long-term neurological improvement can be expected to occur in only about 10% of patients who suffer an initial neurological injury. Notably, the existing evidence shows that in-water TSCI typically occurs in young, healthy males under the age of 30 years who sustain essentially no other associated intracranial or systemic injuries. Most of these injuries result from reckless behaviour. However, involvement of alcohol, no warning signs or depth indicators, and no lifeguard on duty are frequently reported for in-water TSCI.*

*Lifeguards worldwide spend considerable amounts of time practicing complicated techniques on SMR and extrication from difficult aquatic environments believing that these techniques may prevent the development of a secondary TSCI. However, a lack of appropriate, uniform first aid and extrication has been observed (e.g., selection of patients, extrication techniques, and materials used for SMR such as a rigid cervical collar and a hard backboard).*

*Literature regarding in-water TSCI is scarce and mainly constitutes observational studies or biomechanical studies with healthy volunteers considered as low-quality evidence. Due to the rarity of in-water TSCI it is impossible to gather high-quality or even moderate quality evidence. Hence, expert-consensus extrapolating results from the general trauma population may provide the highest level of evidence on the management of in-water TSCI. This calls for a pragmatic handling strategy that is easily understood using equipment that is readily available and believed non-hazardous to the patient and the rescuer. Based on the available body of evidence including the recent consensus- and evidence-based guidelines on SMR of adult trauma patients (see attachment), we aim for a practical consensus guideline on the SMR measures in patients with in-water TSCI.*

**Attachment:**

Maschmann C, Jeppesen E, Rubin MA, Barfod C. New clinical guidelines on the spinal stabilisation of adult trauma patients - consensus and evidence based. Scand J Trauma Resusc Emerg Med. 2019 Aug 19;27(1):77. doi: 10.1186/s13049-019-0655-x. PMID: 31426850; PMCID: PMC6700785.

**Control questions:**

Question 1: “*Literature regarding in-water TSCI is scarce and considered low-quality evidence*” (correct answer: True).

Question 2: “*Due to the rarity of in-water TSCI, it is impossible to gather high-quality or even moderate-quality evidence*” (correct answer: True).

Question 3: “*This study aims for a practical consensus guideline on the SMR measures in patients with in-water TSCI*” (correct answer: True).
