# Supplementary material for: Prehospital guidelines on in-water traumatic spinal injuries for lifeguards and prehospital emergency medical services: an international Delphi consensus study
Source: Scand J Trauma Resusc Emerg Med. 2024 Aug 23;32:76. doi: 10.1186/s13049-024-01249-3 (PMC11344453; doi:10.1186/s13049-024-01249-3)
Supplement: Supplementary file 3 — Additional file 3. [file 13049_2024_1249_MOESM3_ESM.docx]

| **Online Supplement, Appendix C.** Final set of recommendations with consensus levels from Delphi rounds 2 and 3 | | | |
| --- | --- | --- | --- |
| **Final recommendation in Delphi round 3** | **Round 2.1**  **(n=22)** | **Round 2.2**  **(n=21)** | **Round 3**  **(n=21)** |
| **Pre-rescue section** | | | |
| **R1:** It is recommended against using spinal motion restriction when the trauma is due to a mechanism unlikely to cause spinal cord injury. | 95% | NA | 95% |
| **R2:** It is recommended to always alert the available EMS, but this should not delay the rescue. | 86% | NA | 100% |
| **R3:** It is recommended to assess scene safety before attempting a water rescue. This assessment should include the aquatic conditions (such as high surf, fast-moving water, or rocky areas), the level of training and the experience of the lifeguards, the number of lifeguards available and needed, the size of the person, and the equipment available. | 91% | NA | 100% |
| **R4:** It is recommended against using spinal motion restriction in persons suspected of in-water traumatic spinal cord injury in any circumstance with imminent danger of drowning or injury to the lifeguard. | 100% | NA | 100% |
| **R5:** It is recommended against using spinal motion restriction in persons suspected of in-water traumatic spinal cord injury who are unconscious and not breathing normally (suspected cardiac arrest). | 82% | 95% | 95% |
| **Rescue section** | | | |
| **R6:** It is recommended to turn a face-down person suspected of in-water traumatic spinal cord injury immediately and carefully into a face-up position. | 77% | 95% | 90% |
| **R7:** It is recommended to use the AVPU scale to identify an altered level of consciousness in persons suspected of in-water traumatic spinal cord injury. | 91% | NA | 95% |
| **R8:** It is recommended to use the symptom of spinal pain to assess the need for spinal motion restriction in alert persons without a critical ABC problem suspected of in-water traumatic spinal cord injury by asking: “Do you feel pain in your neck or back?”. | 91% | NA | 90% |
| **R9:** It is recommended to use obvious signs of any neurological deficit to assess the need for spinal motion restriction in alert persons without a critical ABC problem suspected of in-water traumatic spinal cord injury by asking: “Can you move your arms and legs?”. | 95% | NA | 90% |
| **R10:** It is recommended to use spinal motion restriction for extrication in alert and oriented persons without a critical ABC problem suspected of in-water traumatic spinal cord injury where self-extrication is impossible. | 95% | NA | 90% |
| **R11:** It is recommended against using a rigid cervical collar in all persons suspected of in-water traumatic spinal cord injury. | 77% | 95% | 95% |
| **R12:** It is recommended to in-line stabilise the head in relation to the thorax with two hands during the extrication if in-water traumatic spinal cord injury is suspected. | 82% | 81% | 86% |
| **R13:** It is recommended to use a floatable, lightweight device that drains water and is appropriate to water conditions to perform spinal motion restriction for extrication of an alert person suspected of in-water traumatic spinal cord injury who cannot perform self-extrication. | 82% | 90% | 95% |
| **R14:** It is recommended against using straps in water unless required for safe extrication. | 82% | 90% | 90% |
| **R15:** It is recommended that one lifeguard trained in spinal motion restriction acts as the team leader and is responsible for the stabilisation of the person's head, the team's safety, supervision, instructions, and coordination. | 95% | NA | 100% |
| **R16:** It is recommended to use at least three persons to perform spinal motion restriction to extricate a person suspected of in-water traumatic spinal cord injury. At least one person should be specifically trained. If the necessary number of persons is not available, do not further delay extrication. | 86% | NA | 81% |
| **R17:** It is recommended to integrate untrained bystanders under the leadership of the lifeguard(s) if there are not enough trained lifeguards available for spinal motion restriction and extrication. | 91% | NA | 90% |
| **R18:** It is recommended against using spinal motion restriction in persons suspected of in-water traumatic spinal cord injury who have NO relevant symptoms. | 91% | NA | 95% |
| **R19:** It is recommended to use self-extrication and self-stabilisation in alert persons suspected of in-water traumatic spinal cord injury who can perform self-extrication and self-stabilisation. | 100% | NA | 95% |
| **Post-rescue section** | | | |
| **R20:** It is recommended to allow an alert person to place him/herself in the most comfortable position during spinal motion restriction on land. | 77% | 95% | 95% |
| **R21:** It is recommended to use the jaw thrust manoeuvre with the head in a neutral position to open the airway in a person suspected of in-water traumatic spinal cord injury who cannot maintain an open airway. | 100% | NA | 95% |
| **Patient selection section** | | | |
| **R22:** It is recommended to treat potentially intoxicated persons in the same way as non-intoxicated persons suspected of in-water traumatic spinal cord injury. | 86% | NA | 86% |
| **R23:** It is recommended to treat persons with distracting injuries in the same way as persons without distracting injuries suspected of in-water traumatic spinal cord injury. | 86% | NA | 86% |
| **R24:** It is recommended to treat persons with language barriers in the same way as persons without language barriers suspected of in-water traumatic spinal cord injury. | 86% | NA | 90% |
| **R25:** It is recommended to treat children in the same way as adults suspected of in-water traumatic spinal cord injury. | 95% | NA | 90% |

Legend: The experts indicated their agreement with each of the recommendations on a 4-point Likert Scale: 1) Strongly disagree, 2) Disagree, 3) Agree, and 4) Strongly agree. The levels shown were calculated as the combined frequencies of “agree” and “strongly agree”; Three categories were defined: 1) Strong consensus (≥85%), 2) Moderate consensus (75-85%), and 3) Weak consensus (<75%). Delphi round 2 was repeated for recommendations with moderate consensus, and recommendations with strong consensus were passed to Delphi round 3. This resulted in 25 recommendations: 24/25 recommendations with strong consensus and 1/25 recommendations (R16) with moderate consensus.
