# Supplementary material for: Prehospital guidelines on in-water traumatic spinal injuries for lifeguards and prehospital emergency medical services: an international Delphi consensus study
Source: Scand J Trauma Resusc Emerg Med. 2024 Aug 23;32:76. doi: 10.1186/s13049-024-01249-3 (PMC11344453; doi:10.1186/s13049-024-01249-3)

| **Online Supplement, Appendix D1.** List of experts who contributed to the generation of the recommendation and the flowchart during all three Delphi rounds | |
| --- | --- |
| **Name** | **Affiliation** |
| Dr. Justin Sempsrott | Lifeguards without borders. United States. ILS-MC; IDRA |
| Ass. Prof. Dr. Peter G. Wernicki | Florida State University School of Medicine; Florida, United States Lifesaving Association; American Red Cross; ILS-MC; ILS-RC; IDRA |
| Ass. Prof. Andreas Claesson | Centre for Resuscitation Science, Department of Clinical Science and Education, Karolinska Institutet, Stockholm, Sweden; IDRA |
| Mr. Josh Carmine | Drowning Prevention Auckland, New Zealand; IDRA |
| Dr. Patrick Morgan | Extreme Environments Laboratory, University of Portsmouth, Portsmouth UK; HM Coastguard, Southampton UK; Surf Lifesaving GB, Exeter, UK; ILS-MC; IDRA |
| Mrs. Monica Fernandez-Robles | Baby Survival Swim, Guatemala; IDRA |
| Mr. Leonardo Springer | ISEC Lisboa-TGRAF, Lisbon, Portugal; ULisboa FBAUL-CIEBA; ILS-RC; IDRA |
| Mg. Manino Leonardo Andres | Sociedad Argentina de Medicina Pre Hospitalaria CPR Committee, Rosario City, Argentina; ILS-MC; IDRA |
| Dr. Mohamed Saleh | EDLF EGYPT, Cairo, Egypt; ILS-MC |
| Mr. Alex Kam Hung Liu | The Hong Kong Life Saving Society, Wnachai, Hong Kong; ILC-RC |
| Mr. Adrian Mayhew | Surf Life Saving Great Britain, Exeter, United Kingdom; ILS-RC |
| Mr. Riley Huntley | Royal Life Saving Society Canada; University of British Columbia, Vancouver, Canada; IDRA |
| Dr. Allart M. Venema | Department of Anesthesiology, University Medical Center Groningen, University of Groningen, Groningen, The Netherlands; IDRA |
| Mr. Ramses Marti Biosca | Sistema Emergències Mèdiques SEM, Public Prehospital Emergency Service of Catalonia, Spain; IDRA |
| Dr. Cody Dunne | University of Calgary, Alberta, Canada; ILS-MC; IDRA |
| Dr. Gary Payinda | Surf Life Saving New Zealand/SLSNZ; New Zealand Resuscitation Council/NZRC; ILS-MC |
| Dr. Eric Tellier | INSERM, Université de Bordeaux U1219 ISPED Bordeaux population health research Bordeaux, France; IDRA |
| Prof. Silvia Aranda-García | GRAFAIS Research Group, Institut Nacional d'Educació Física de Catalunya/INEFC, University of Barcelona, Barcelona, Spain; IDRA |
| Prof. Ismael Sanz Arribas | Departamento de Educación Física, Deporte y Motricidad Humana, Facultad de formación de Profesorado y Educación de la Universidad Autónoma de Madrid, Madrid, Spain; Real Federación Española de Salvamento y Socorrismo; ILS-MC |
| Dr. Natalie Hood | Monash Medical Centre, Melbourne, Australia; Surf Life Saving Australia; ILS-MC |

All authors marked with “ILS-MC” or “ILS-RC” are affiliated with the International Life Saving Federation, Leuven, Belgium. All authors marked with “IDRA” are affiliated with the International Drowning Researchers' Alliance, Kuna, Idaho.

Abbreviations: IDRA = International Drowning Researchers’ Alliance; ILS = International Life Saving Federation; ILS-MC = International Life Saving Federation Medical Committee; ILS-RC = International Life Saving Federation Rescue Commission.

**Online Supplement, Appendix D2.** The geographical distribution of the experts listed in Appendix D1.


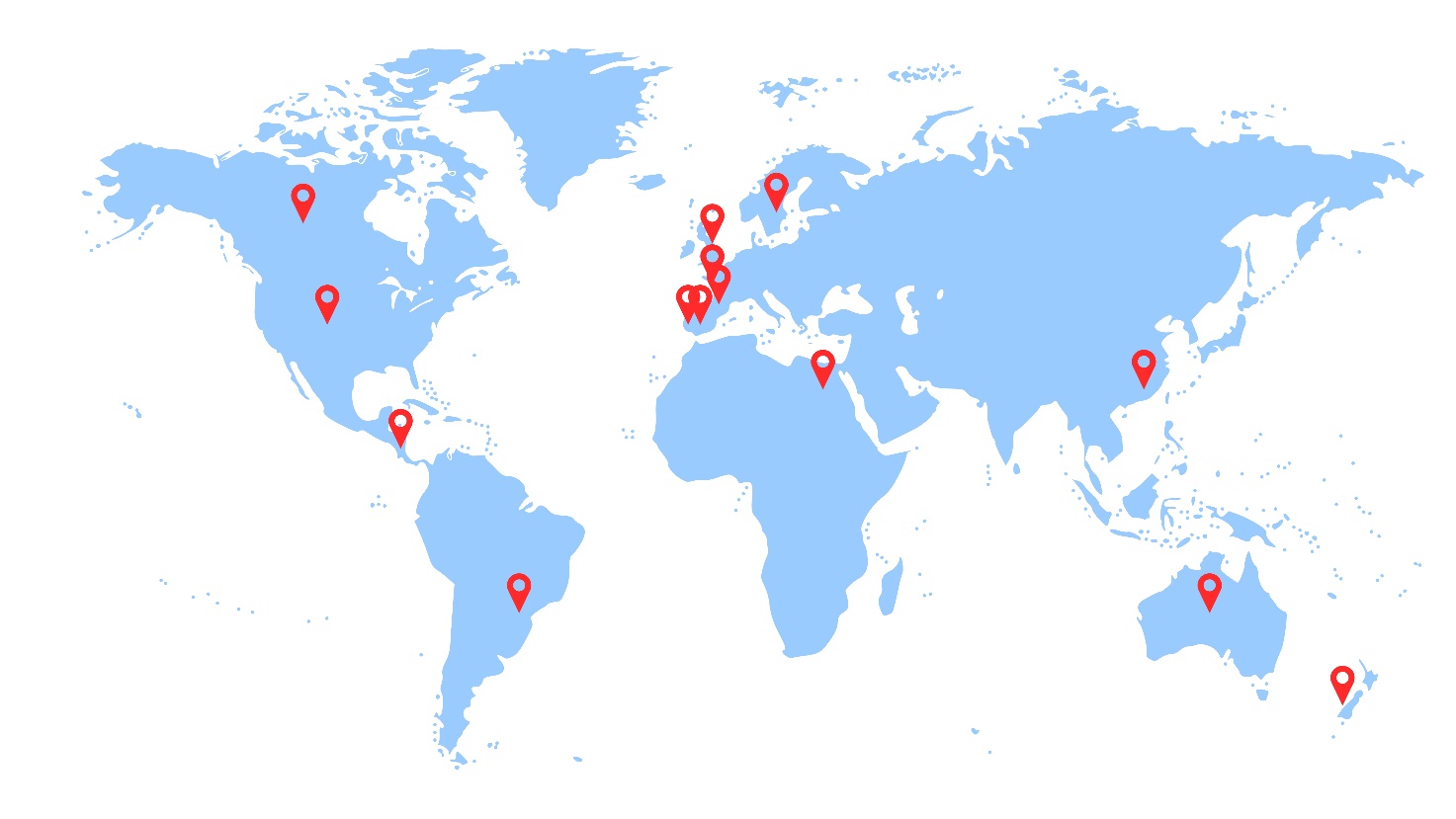

Supplement: Supplementary file 4 — Additional file 4. [file 13049_2024_1249_MOESM4_ESM.docx]
